# A Novel Peptoid Hybrid of Alpha-Calcitonin Gene-Related Peptide (α-CGRP) Ameliorates Cardiac Remodeling in Pressure Overload-Induced Heart Failure

**DOI:** 10.3390/cells14201580

**Published:** 2025-10-11

**Authors:** Sarah Deloach, Ambrish Kumar, Emily Ruggiero, Ryan Ball, Kamryn Gleason, Jason Kubinak, Donald J. DiPette, Jay D. Potts

**Affiliations:** 1 Department of Biomedical Engineering, College of Engineering and Computing, University of South Carolina, Columbia, SC 29208, USA; sdeloach@email.sc.edu (S.D.); kamryng@email.sc.edu (K.G.); 2 Department of Cell Biology and Anatomy, School of Medicine, University of South Carolina, Columbia, SC 29208, USA; ambrish.kumar@uscmed.sc.edu (A.K.); emily.ruggiero@uscmed.sc.edu (E.R.); 3 Department of Pathology, Microbiology, and Immunology, School of Medicine, University of South Carolina, Columbia, SC 29208, USA; ryan.ball@uscmed.sc.edu (R.B.); jason.kubinak@uscmed.sc.edu (J.K.); 4 Department of Internal Medicine, School of Medicine, University of South Carolina, Columbia, SC 29208, USA; donald.dipette@uscmed.sc.edu

**Keywords:** calcitonin gene-related peptide (CGRP), cardiovascular diseases, congestive heart failure, neuropeptide, pressure-overload heart failure, therapeutic efficacy, transverse aortic constriction (TAC)

## Abstract

α-CGRP (alpha-calcitonin gene-related peptide) is a vasoactive and anti-inflammatory neuropeptide that is cardioprotective in transverse aortic constriction (TAC)-induced pressure overload heart failure (HF) models. Our previous investigations established that a peptoid modification of α-CGRP, termed NMEG-CGRP, prevented left ventricular (LV) dysfunction and remodeling when administered subcutaneously every other day for 28 days, starting two days post-TAC surgery (termed prevention study). Here, we determined whether NMEG-CGRP would be cardioprotective when administered after the development of LV dysfunction secondary to TAC surgery (termed treatment study). Starting 15 days post-sham or TAC surgery, we administered NMEG-CGRP (3.6 mg/kg/mouse) subcutaneously every other day for 28 days in mice assigned to treatment groups. In vivo assessments included weekly electrocardiography to evaluate cardiac function and blood sampling for immunophenotyping. On Day 45, mice were euthanized, and hearts were collected for gross, histological, and biochemical analyses. Compared to sham-operated mice, TAC mice exhibited decreased LV ejection fraction and increased hypertrophy, dilation, fibrosis, apoptosis, and oxidative stress. In contrast, TAC mice treated with NMEG-CGRP demonstrated significant improvements in cardiac function and cellular and biochemical parameters when compared to TAC mice. These findings demonstrate the therapeutic potential of NMEG-CGRP in the treatment of established cardiovascular dysfunction and its progression in pressure overload-induced HF.

## 1. Introduction

Alpha-calcitonin gene-related peptide (α-CGRP), a 37-amino-acid neuropeptide, is the most potent vasodilator known to date and exerts positive chronotropic and inotropic effects [1,2,3]. Previous studies from our laboratory and others have established that α-CGRP is protective in a variety of cardiovascular diseases, including heart failure (HF), myocardial infarction, ischemic–reperfusion injury, and experimental hypertension [4,5,6,7,8,9,10,11,12,13]. α-CGRP confers its protective effects through anti-hypertrophic, anti-fibrotic, anti-apoptotic, antioxidant, and anti-inflammatory mechanisms [14,15,16]. In addition to exerting a direct effect on vasculature, α-CGRP release is thought to be the driving force behind the cardioprotective mechanisms of sodium glucose transporter 2 inhibitors, which are currently prescribed to treat HF [17]. We have demonstrated that α-CGRP administration lowers blood pressure (BP) in both normotensive and hypertensive animal models, as well as in humans [18,19,20,21]. Furthermore, we have shown in a transverse aortic constriction (TAC)-induced pressure overload HF model that genetically engineered α-CGRP knockout (KO) mice develop greater left ventricular (LV) dysfunction, hypertrophy, dilation, fibrosis, and mortality compared to wild-type mice [9]. Additionally, we established that long-term exogenous delivery of native α-CGRP via osmotic mini-pumps was cardioprotective in TAC-induced pressure overload HF in wild-type mice [22]. Administration of native α-CGRP for 28 days preserved cardiac function and structure and reduced myocardial apoptosis, fibrosis, and oxidative stress in the LVs of TAC mice. Similarly, two separate studies confirmed that infusion of either native α-CGRP or an acylated α-CGRP analog (t_1/2_ ≥ 7 h) significantly improved cardiac function in rodent models of hypertension and HF [12,23]. Collectively, these findings validate that α-CGRP is a promising therapeutic candidate for the treatment of multiple cardiovascular diseases, such as HF, myocardial ischemia and infarction, and hypertension.

Unfortunately, the therapeutic potential for α-CGRP is hindered by its extremely short half-life (t_1/2_ ≈ 5.5 min in human plasma) and the impracticality of using implanted osmotic mini-pump delivery systems or continuous intravenous infusion in humans with chronic cardiovascular disease [24]. Novel approaches to increase peptide bioavailability in serum are urgently needed, which led our laboratory to develop long-acting α-CGRP analogs. In our most recent approach, we chemically synthesized human α-CGRP analogs by linking two *N*-methoxyethyl glycine (NMEG) peptoid monomers at either the N-terminus (termed NMEG-CGRP) or C-terminus (termed CMEG-CGRP). A peptoid monomer is an *N*-substituted glycine molecule analogous to an α-amino acid, except that its side chain (R-group) is attached to the nitrogen atom rather than the α-carbon atom. The side chain (R-group) substitution makes peptoids more protease-resistant while retaining the native peptide’s physiochemical properties [25,26,27]. Currently, peptoids serve as versatile molecular tools in biochemistry and biophysics and are becoming attractive candidates for therapeutic and diagnostic applications [28,29,30]. To date, such peptoids have demonstrated bioactivity as protein mimics and as replacements for small-molecule pharmacological agents [31,32,33]. Several peptoids have been synthesized that exhibit antibacterial, antifungal, antiparasitic, and anti-Alzheimer’s disease activity [30,34,35,36,37].

We previously demonstrated that of the two peptoids, NMEG-CGRP is pharmacodynamically bioactive (lowers blood pressure following subcutaneous administration) while CMEG-CGRP is not. Additionally, we demonstrated in vitro that NMEG-CGRP and CMEG-CGRP were non-toxic to myocardial cells and more protease-resistant than native CGRP, while still maintaining bioactivity [38]. Using a murine TAC-induced pressure overload HF model, we then determined whether NMEG-CGRP administration every other day for 28 days, starting two days post-TAC, prevented the development of cardiac dysfunction and remodeling (termed prevention study) [38]. We observed an increase in ejection fraction, in addition to significant decreases in cardiac hypertrophy, LV fibrosis, oxidative stress, apoptosis, and macrophage infiltration [38]. Therefore, we established that NMEG-CGRP prevented the development of cardiac dysfunction and remodeling through anti-hypertrophic, anti-fibrotic, anti-apoptotic, and antioxidant mechanisms [38].

Here, we determined whether NMEG-CGRP administration could ameliorate cardiac dysfunction and remodeling after the establishment of LV dysfunction (termed treatment study). Additionally, we examined cardiac remodeling through elastin morphogenesis and explored the immunomodulatory role of NMEG-CGRP by analyzing tissue and circulating immune cell populations. Cardiac remodeling is driven by circulating leukocytes which can drive either pro- or anti-inflammatory effects on inflamed cardiac tissue [39]. Tissue-resident macrophages are key modulators of both the innate and adaptive immune responses which serve to maintain cardiac homeostasis [40]. The immunomodulatory role of CGRP has been explored in preclinical models of diabetes, sepsis, and gastrointestinal diseases such as Crohn’s disease, colitis, and inflammatory bowel disease [41,42,43,44,45,46]. CGRP and its receptors are distributed throughout the lymphoid system and can be produced by or bind to several immune cell subsets including T cells, B cells, dendritic cells (DCs), mast cells, and macrophages [47]. CGRP can induce pro- or anti-inflammatory activities in these cell subsets, with anti-inflammatory and regenerative phenotypes identified in macrophages and neutrophils [47,48,49]. Our findings indicate that subcutaneous administration of NMEG-CGRP, starting 15 days post-TAC with established LV dysfunction, ameliorates cardiac remodeling at the physiological and cellular level. Furthermore, NMEG-CGRP can influence circulating and tissue-resident immune cell populations to shift toward anti-inflammatory phenotypes. These results highlight the therapeutic potential of using α-CGRP analogs as an innovative approach to treating cardiovascular diseases.

## 2. Materials and Methods

### 2.1. Pressure Overload-Induced Heart Failure Model

All experiments were performed in accordance with the National Institutes of Health (NIH) guidelines and with approval from the University of South Carolina Institutional Animal Care and Use Committee (IACUC). Eight-week-old male C57BL/6 mice were purchased from Charles River Laboratories (Wilmington, MA, USA) and housed in the institutional animal facility on a 12 h light–dark cycle with free access to standard food and water.

Pressure overload heart failure was induced in mice using a transverse aortic constriction (TAC) surgery [9,11,22]. Briefly, mice were anesthetized under 1–2% isoflurane gas, and the chests were opened through the suprasternal notch. A 7-0 polypropylene suture was passed under the aortic arch between the left common carotid and innominate arteries and tied against a 27-gauge needle. After tying the knot, the needle was removed, and the chest was closed using a 6-0 suture. Mice recovered on a 37 °C heating pad and buprenorphine (0.1 mg/kg/mouse) was administered subcutaneously for postoperative care. Sham-operated mice underwent the same procedure without aortic constriction. Mice were randomly divided into four groups after surviving the initial surgery: Sham (n = 8), Sham+NMEG-CGRP (n = 7), TAC (n = 8), and TAC+NMEG-CGRP (n = 9). Fifteen days post-TAC, NMEG-CGRP (3.6 mg/kg/mouse) was administered subcutaneously every other day for 28 days in Sham+NMEG-CGRP and TAC+NMEG-CGRP groups. At the end of the experiment (Day 44), mice were euthanized, and hearts were collected, weighed, and photographed. Hearts were bisected transversely at the mid-ventricular level and the basal portion was fixed in 4% paraformaldehyde (PFA)/PBS (pH 7.4). The apical portion was further bisected transversely, and the basal-ventricular segment was fixed in 2% glutaraldehyde/2% PFA/PBS (pH 7.4), the mid-ventricular segment was fixed in RNA*later*™ (Invitrogen, Carlsbad, CA, USA), and the apical segment was snap-frozen in liquid N_2_ and stored at −80 °C for biochemical analyses.

A Vevo 3100 High-Resolution Imaging System (FUJIFILM VisualSonics, Toronto, ON, Canada) was used to perform short-axis 2D echocardiography two days before TAC surgery (Day 0) followed by weekly assessments post-TAC until Day 44 (Table S1) [9,22]. Mice were anesthetized under 2% isoflurane and heart rate was maintained at 450 ± 25 beats per minute. Short-axis B- and M-mode 2D echocardiograms were recorded through the anterior and posterior LV walls at the level of the papillary muscle. Ejection fraction (EF) was calculated using the Vevo LAB analysis software (FUJIFILM VisualSonics, Toronto, ON, Canada) [9,22].

### 2.2. Immunohistochemistry

Paraformaldehyde-fixed paraffin-embedded left ventricle (LV) sections (5 µm thick) were deparaffinized and rehydrated using xylene and graded ethanol (100%, 95%, and 70%) washes. Slides were stained with a Texas Red-X conjugated wheat germ agglutinin (WGA; Invitrogen, Carlsbad, CA, USA) to measure cardiomyocyte cross-sectional area, Sirius Red/Fast Green (Chondrex, Woodinville, WA, USA) to quantify fibrosis, a DeadEnd™ fluorometric TUNEL system (Promega, Madison, WI, USA) to detect apoptosis, and an Alexa Fluor 546-conjugated MAC387 antibody (Santa Cruz Biotechnology, Dallas, TX, USA) to assess macrophage infiltration according to the manufacturer’s protocols. LV sections were imaged using an EVOS FL Auto Cell Imaging System (Invitrogen, Carlsbad, CA, USA) and quantified using Fiji [38]. Additionally, Cellpose 2.0 was used to segment cardiomyocytes and QuPath 0.5.0 was used to measure fibrosis prior to quantification in Fiji [50,51,52].

Immunofluorescence staining was performed on paraformaldehyde-fixed paraffin-embedded LV sections (5 µm thick) that were deparaffinized and rehydrated as previously described. Antigen unmasking was performed by boiling slides in 10 mM sodium citrate buffer (pH 6.0) for 30 min. After permeabilization with 0.2% Triton X-100/PBS for 10 min, LV sections were blocked with 10% IgG-free-BSA/PBS (Jackson ImmunoResearch Laboratories, West Grove, PA, USA) and incubated with primary antibodies overnight at 4 °C. The primary antibodies used were cleaved caspase-3 (Cell Signaling Technology, Danvers, MA, USA), 4-hydroxy-2-noneal (4-HNE; Abcam Inc, Cambridge, UK), and 8-hydroxy-2′-deoxyguanosine (8-OHdG; Santa Cruz Biotechnology, Dallas, TX, USA) at 1:100 dilutions. Alexa Fluor 488 or Alexa Fluor 546-conjugated secondary antibodies (Invitrogen, Carlsbad, CA, USA) were added to detect protein signals and DAPI (4′, 6-diamidino-2-phenylindole; Sigma-Aldrich, St. Louis, MO, USA) was added to detect nuclei. After mounting with antifade DABCO (1, 4-diazobicyclo-2,2,2-octane; Sigma-Aldrich, St. Louis, MO, USA) mounting media, LV sections were imaged using an EVOS FL Auto Cell Imaging System (Invitrogen, Carlsbad, CA, USA). For each group, 35 regions of the LV were captured, and images were distributed evenly across groups (≈3–5 images per mouse). Images were imported into Fiji and quantified using positive cell counts for TUNEL and cleaved caspase-3 staining and integrated density for 8OHdG and 4-HNE staining [38]. Cells were considered positive if a fluorescent signal was observed in the nucleus. To measure integrated density, images were converted to 8-bit, and an automatic threshold was used to isolate regions of interest (ROI). The scale was set by measuring the image’s existing scale bar and integrated density values were measured for ROIs. Staining, image acquisition, and quantification was performed by an experimenter that was blinded to which mouse belonged to each group.

### 2.3. Flow Cytometry

Whole blood (≤50 µL) was collected from the saphenous vein two days before TAC surgery (Day 0), followed by weekly assessments post-TAC until Day 44. Blood was collected using heparin-coated capillary tubes and incubated with 2 mL of 1× RBC lysis buffer (BioLegend, San Diego, CA, USA) for 15 min at room temperature (RT). Samples were centrifuged at 350 RCF for 10 min at 4 °C, resuspended in 200 µL of column buffer (1× HBSS, 1 M HEPES, 5% FBS, 0.5 M EDTA), and centrifuged again for 5 min at 4°C. The pellet was resuspended in 100 µL of column buffer containing 1:500 TruStain FcX™ PLUS (BioLegend, San Diego, CA, USA) for 10 min at RT. After centrifugation at 350 RCF for 3 min at 4 °C, samples were resuspended in 200 µL of 1× PBS and centrifuged. Following this, samples were incubated in 100 µL of 1× PBS containing 1:500 Zombie Aqua™ Fixable Viability dye (BioLegend, San Diego, CA, USA) for 20 min at RT, followed by centrifugation and resuspension in 200 µL of column buffer. Samples were incubated with 100 µL of antibody master mix (Table 1), each antibody at a 1:100 dilution in column buffer, for 20 min at RT. Samples were centrifuged at 350 RCF for 3 min at 4 °C and resuspended into 200 µL of column buffer. Samples were centrifuged once more at 350 RCF for 3 min at 4 °C, resuspended into 300 µL of column buffer, and transferred to round bottom polystyrene test tubes containing 300 µL of 4% PFA/PBS. Data acquisition was performed on a BD FACSymphony A5 Cell Analyzer (BD Biosciences, San Jose, CA, USA), and data analysis was performed using FlowJo software (version 10.10.0; BD Biosciences, San Jose, CA, USA). Using single stained controls, a compensation matrix was generated and applied to all sample data files. Manual gating was performed to exclude doublets, debris, and dead cells followed by the identification of immune cell populations (CD45+). From CD45+ cell populations, myeloid and lymphoid subsets were characterized (Figure S1). Staining, data acquisition, and gating was performed by an experimenter that was blinded to which mouse belonged to each group.

### 2.4. Elastin Morphology

Paraformaldehyde-fixed paraffin-embedded sections (6 µm thick) of the distal aortic arch were deparaffinized and rehydrated as previously described. Sections were stained with modified Masson’s Trichrome (Polysciences, Warrington, PA, USA) with the Biebrich Scarlet-Acid Fuchsin solution omitted to enhance elastin visualization. Sections were imaged using a Revolve Microscope (Discover Echo, San Diego, CA, USA) and preprocessed using Fiji [50]. In Fiji, elastin fibers were manually traced using the paintbrush tool, thresholded, and converted to binary images for structural analysis (Figure S2). Binary tracings were exported to Amira software (Thermo Fisher Scientific, Waltham, MA, USA) for individual elastin fiber quantification using the Fiber Tracking module. Length and tortuosity were assessed for individual elastin fibers. To ensure data quality and consistency, fibers shorter than 100 µm in length and those with tortuosity values greater than 10 were excluded from analysis. Additionally, animals which showed an open aortic lumen and near-linear fiber orientation (tortuosity ~ 1), were excluded from the final data set due to anatomical inconsistencies. Because of these strict requirements, the sample size of each group was reduced. Staining, image acquisition, and quantification were performed blinded to which section belonged to each group.

### 2.5. Statistical Analysis

Statistical analysis was performed in Prism 10 (version 10.0.2; GraphPad Software, Boston, MA, USA). Data sets were tested for normal distribution using the Shapiro–Wilk test. For data collected at a single time point, normally distributed values were analyzed using one-way analysis of variance (ANOVA) followed by Tukey’s multiple comparisons test, while non-normally distributed values were analyzed using the Kruskal–Wallis test followed by Dunn’s multiple comparison test. For data collected longitudinally, normally distributed values were analyzed using two-way ANOVA followed by Tukey’s multiple comparison test. For non-normally distributed longitudinal data, a mixed-effects analysis followed by Tukey’s multiple comparison test was performed. Data sets were reported as the mean ± SEM and *p*-values < 0.05 were considered statistically significant.

## 3. Results

### 3.1. NMEG-CGRP Improved Left Ventricular Systolic Function After the Onset of HF Symptoms

**Figure 1:**
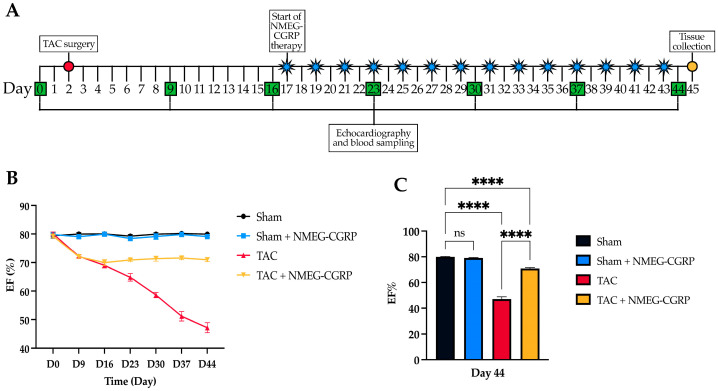
(**A**) Diagram of the experimental timeline showing blood sampling, echocardiography, NMEG-CGRP treatment schedule. Echocardiograms and blood sampling began two days prior to TAC surgery (Day 0/D0). Following TAC surgery (D2), echocardiograms and blood sampling were performed weekly (D9, D16, D23, D30, D37, D44) until study termination (D45). NMEG-CGRP therapy (3.6 mg/kg/mouse) began at D17 and was administered every other day (D19, D21, D23, D25, D27, D29, D31, D33, D35, D37, D39, D41, D43) until study termination (D45). (**B**) Longitudinal echocardiographic measurements of ejection fraction (EF) across experimental groups. (**C**) One-way analysis of variance (ANOVA) of EF across experimental groups on Day 44. ns = not significant, **** *p* < 0.0001.

Echocardiograms were collected two days prior to TAC surgery, followed by weekly assessments starting 7 days post-TAC until study termination. After 15 days post-TAC, mice belonging to Sham+NMEG-CGRP (n = 7) and TAC+NMEG-CGRP (n = 9) groups began receiving subcutaneous injections of NMEG-CGRP (3.6 mg/kg/mouse) on alternate days over the course of 28 days (Figure 1A). Short-axis 2D echocardiography was performed to calculate ejection fraction (EF) as a measure of cardiac function (Figure 1B). Our results demonstrated that TAC significantly reduced EF over time, whereas the administration of NMEG-CGRP halted the progression of left ventricular (LV) dysfunction (Figure 1C). We observed no differences between the Sham (n = 7) and Sham+NMEG-CGRP groups (n = 8), and no abnormalities or adverse effects were noted in NMEG-CGRP-treated mice.

### 3.2. NMEG-CGRP Reduces Cardiac Remodeling After the Onset of HF Symptoms

**Figure 2:**
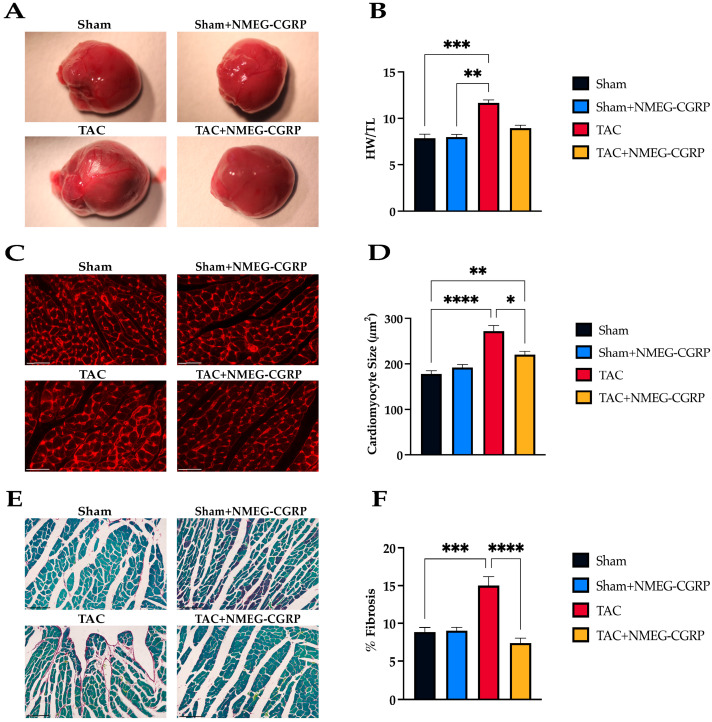
(**A**) Representative images of gross heart anatomy across experimental groups. (**B**) Kruskal–Wallis test of the heart weight to tibia length ratio. (**C**) Representative images of left ventricle (LV) sections stained with Texas Red-X conjugated wheat germ agglutinin (scale bar–50 µm). (**D**) Kruskal–Wallis test of cross-sectional cardiomyocyte area (µm^2^). (**E**) Representative images of LV sections stained with Sirius Red/Fast Green (scale bar–75 µm). (**F**) Kruskal–Wallis test of LV collagen content. * *p* ≤ 0.05, ** *p* ≤ 0.01, *** *p* ≤ 0.001, **** *p* ≤ 0.0001.

Pressure overload causes structural changes to the heart though increased inflammation and fibroblast proliferation [9]. Hearts were collected, weighed, photographed, and processed for histological assessment. The heart weight to tibia length ratio (HW/TL) was used to quantify cardiac hypertrophy. Our results showed that gross heart size and HW/TL ratio were larger in TAC mice when compared to sham mice, whereas heart size and HW/TL ratio were reduced in TAC mice treated with NMEG-CGRP (Figure 2A,B). To further assess hypertrophy, left ventricular (LV) sections were stained with WGA, imaged, and cardiomyocyte cross-sectional area was quantified using Cellpose 2.0 and Fiji (Figure 2C) [50,51]. A significant increase in cardiomyocyte size (µm^2^) was observed in the TAC group when compared to the Sham group, while a decrease was observed between the TAC and TAC+NMEG-CGRP groups (Figure 2D). In addition, LV fibrosis was assessed using a Sirius Red/Fast Green collagen staining kit and quantified in QuPath 0.5.0 (Figure 2E) [52]. An increase in fibrosis was observed in TAC mice when compared to sham mice. In contrast, fibrosis was significantly reduced in TAC mice treated with NMEG-CGRP (Figure 2F).

### 3.3. NMEG-CGRP Ameliorates Increased Apoptosis and Oxidative Stress After the Onset of HF Symptoms

**Figure 3:**
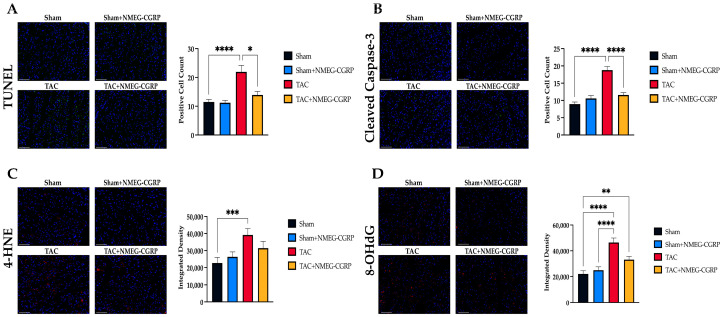
(**A**) Representative images of LV sections across all experimental groups stained using a fluorometric TUNEL kit (scale bar–100 µm), alongside a Kruskal–Wallis test of TUNEL-positive cell counts. (**B**) Representative images of LV sections stained with a primary antibody against cleaved caspase-3 paired with Alexa Fluor 488 (scale bar–100 µm), alongside a one-way ANOVA of cleaved caspase-3-positive cell counts. (**C**) Representative images of LV sections stained with a primary antibody against 4-hydroxy-2-nonenal (4-HNE) paired with Alexa Fluor 546 (scale bar–100 µm), alongside a Kruskal–Wallis test of integrated density. (**D** )Representative images of LV sections stained with a primary antibody against 8-hydroxy-2′-deoxyguanosine (8-OHdG) paired with Alexa Fluor 546 (scale bar–100 µm) alongside a Kruskal–Wallis test of integrated density. * *p* ≤ 0.05, ** *p* ≤ 0.01, *** *p* ≤ 0.001, **** *p* ≤ 0.0001.

Apoptosis and oxidative stress drive cardiac remodeling in heart failure through reduced cardiomyocyte contractility and mitochondrial dysfunction [9]. To assess apoptotic cell death, LV sections were separately stained using a fluorometric TUNEL kit and a primary antibody against cleaved caspase-3 (Figure 3A,B). After staining, LV sections were imaged, and positive cell counts were performed in Fiji [50]. Our results showed that the number of TUNEL-positive and cleaved caspase-3-positive cells was significantly increased in the TAC group when compared to the Sham group (Figure 3A,B). In contrast, positive cell counts were reduced in the TAC+NMEG-CGRP group when compared to the TAC group. To assess oxidative stress, LV sections were separately stained with primary antibodies against 4-hydroxy-2-nonenal (4-HNE) and 8-hydroxy-2′-deoxyguanosine (8-OHdG) (Figure 3C,D). After staining, LV sections were imaged, and integrated density was quantified in Fiji [50]. Our results showed that the integrated densities of both 4-HNE and 8-OHdG were significantly increased in the LV sections of TAC mice, whereas integrated density were reduced in TAC mice treated with NMEG-CGRP.

### 3.4. NMEG-CGRP Protects Aortic Elastin Fiber Tortuosity

**Figure 4:**
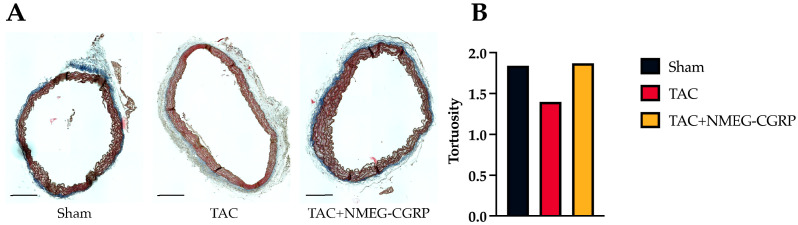
(**A**) Representative images of distal aortic arch sections from Sham, TAC, and TAC+NMEG-CGRP groups stained using modified Masson’s Trichrome (scale bar–300 µm). (**B**) Average tortuosity across Sham, TAC, and TAC+NMEG-CGRP groups.

Vascular remodeling is induced by changes in hemodynamics that lead to decreased vascular wall integrity, increased stiffness, and increased aortic diameter [53]. To assess the vascular morphology, sections of the distal aortic arch were stained with modified Masson’s Trichrome, imaged, and preprocessed in Fiji [50] (Figure 4A). Binary traces of the elastin fibers were exported from Fiji and quantified in Amira using the Fiber Tracking module (Figure S2A,B). Our results showed a decrease in elastin fiber tortuosity in the TAC group when compared to the Sham group (Figure 4B). In contrast, fiber tortuosity in the TAC+NMEG-CGRP group was comparable to that in the Sham group. These findings imply that while TAC reduces fiber tortuosity and rigidity, CGRP treatment appears to reverse this effect, potentially preserving or restoring fiber integrity.

### 3.5. NMEG-CGRP Acts as an Anti-Inflammatory Immunomodulator at the Circulatory and Tissue Levels

**Figure 5:**
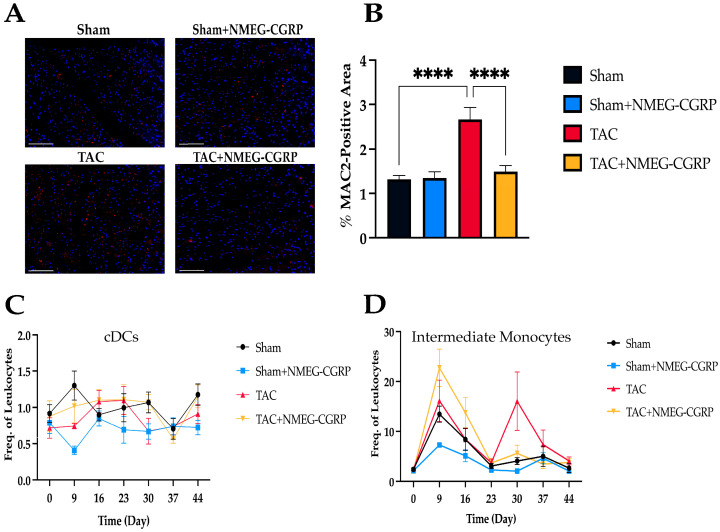
(**A**) Representative images of left ventricle (LV) sections across all experimental groups stained using Alexa Fluor 546-conjugated MAC387 (scale bar–100 µm). (**B**) One-way ANOVA of LV macrophage infiltration (**C**) Changes in transitional monocyte frequency over time across experimental groups (**D**) Changes in activated B cell and CD4+ T cell frequency over time across experimental groups. **** *p* ≤ 0.0001.

Circulating and tissue-resident pro-inflammatory immune cells are hallmarks of heart failure and correlate with disease severity and outcome [39]. To assess cardiac inflammation, LV sections were stained with an Alexa Fluor 546-conjugated MAC387 antibody, imaged, and quantified in Fiji (Figure 5A) [50]. Our results showed that TAC mice had increased macrophage infiltration when compared to sham mice, whereas macrophage infiltration was decreased in TAC mice treated with NMEG-CGRP (Figure 5B). To assess changes in circulating immune cell populations, we collected weekly blood samples which were stained with 20 fluorescently labeled antibodies (Table 1) and 11 immune cell subsets were quantified (Figures S1 and S3A–K). Mixed-effects analysis was used to evaluate time and treatment effects and time (x) treatment interactions. Significant main effects of time were observed across all immune cell populations except for conventional dendritic cells (cDCs), whereas significant main effects of treatment were observed in transitional monocytes (Figure 5C). Significant time x treatment interactions were observed in activated B cells and CD4+ T cells (Figure 5D). The frequency of transitional monocytes remained elevated from Day 23 (D23) to D44 in TAC mice, whereas they remained infrequent across Sham, Sham+NMEG-CGRP, and TAC+NMEG-CGRP groups (Figure 5C). The frequency of activated B cells and CD4+ T cells in TAC mice treated with NMEG-CGRP exhibited similar fluctuation patterns from D23 to D44.

## 4. Discussion

Studies from our laboratory and other research groups have established that alpha-calcitonin gene-related peptide (α-CGRP), in both native and analog forms, attenuates cardiac remodeling in cardiovascular diseases including heart failure (HF), myocardial infarction, and experimental hypertension [4,5,6,7,8,9,10,11,12,13,14]. In patients with stable angina pectoris, intracoronary infusion of α-CGRP induced coronary vasodilation and delayed the onset of myocardial ischemia [54,55]. Additionally, in patients with HF, acute intravenous infusion of α-CGRP improved myocardial contractility and decreased systemic arterial pressure [20,56]. Our previous work has established that long term administration of α-CGRP via osmotic mini-pumps is cardioprotective at physiological and cellular levels in transverse aortic constriction (TAC)-induced pressure overload HF [22]. Another study using α-CGRP knockout (KO) mice showed that α-CGRP delivery through osmotic mini-pumps corrected the adverse effects of hypertension in these KO mice [23].

We previously demonstrated that NMEG-CGRP administered subcutaneously every other day for 28 days, starting two days post-TAC surgery (termed prevention study), prevented or attenuated the development of heart failure pathophysiology [38]. Those studies established that NMEG-CGRP is non-toxic, protease resistant, and bioactive [38]. Additionally, we showed that subcutaneous administration of NMEG-CGRP slowed adverse cardiac remodeling. We observed an increased ejection fraction (EF), and decreased fibrosis, hypertrophy, apoptosis, oxidative stress, and tissue inflammation in TAC mice treated with NMEG-CGRP [38]. In our current study, we showed NMEG-CGRP administration starting 15 days post-TAC was also effective in ameliorating cardiac remodeling in established left ventricular (LV) dysfunction secondary to TAC surgery (termed treatment study). Additionally, we examined elastin tortuosity and integrity and changes in circulating and tissue-resident immune cell populations.

Our findings demonstrated that NMEG-CGRP is protective against cardiac remodeling in a murine TAC-induced pressure overload HF model after the onset of LV dysfunction. Similarly to our prevention study, administration of NMEG-CGRP every other day for 28 days, starting 15 days post-TAC, attenuated cardiac remodeling based on increased ejection fraction (EF). Upon administration of NMEG-CGRP in TAC mice, EF was immediately preserved and remained stable for the duration of the study (until Day 44/D44). Furthermore, gross heart size and cardiomyocyte hypertrophy were reduced, and interstitial fibrosis was markedly reduced, similar to that observed in our earlier prevention study [38]. Furthermore, it has been shown that CGRP-derived from cardiac fibroblasts suppressed TGFβ induced cardiac fibrosis, most likely through a NF-KB signaling pathway [15]. Our results may point to an anti-fibrotic mechanism that functions through a similar pathway, but further investigation is required. Previous studies from our group showed that treatment of CGRP immediately after TAC surgery reduced apoptosis and decreased oxidative stress [11,22,38]. Here, we demonstrated that treatment initiated after the onset of HF similarly produced a reduction in LV apoptosis and oxidative stress in TAC mice treated with NMEG-CGRP.

In this study, we further explored the influence of pressure overload and NMEG-CGRP treatment on elastin morphology and on circulating and tissue-resident immune cell populations. Increased hemodynamic load has been shown to reduce elastin tortuosity and increase artery stiffness [53]. This reduction in elastin tortuosity was also observed in our TAC group but returned to normal geometry in the TAC+NMEG-CGRP group. This may be attributed to the preservation of hemodynamics that occurs upon NMEG-CGRP administration; however, anti-fibrotic and anti-inflammatory mechanisms could also be responsible. Transitional monocytes are circulating monocyte precursors that evolve into inflammatory monocytes and tissue-resident macrophages under inflammatory signaling cascades [57]. Transitional monocyte levels were elevated in the TAC group for the majority of the experimental timeline, whereas by Day 23 levels in the TAC+NMEG-CGRP group were reduced to baseline. Both of these markers play a role in modulating inflammatory response, and this line of evidence was further supported by a decrease in macrophage infiltration observed in LV sections from the TAC+NMEG-CGRP group [57,58]. This indicates that NMEG-CGRP may play a role in dampening the inflammatory response. Additionally, we observed parallel fluctuation patterns between activated B cells and CD4+ T cells. Interactions between T and B cells play an important role in cardiac homeostasis and injury response and have been shown to induce pro- or anti-inflammatory mechanisms in several cardiovascular disease models [46]. Our blood immunophenotyping results showed significant changes in the immune cell population across all of our experimental groups over time, and that TAC mice treated with NMEG-CGRP exhibited a reduction in the transitional monocyte frequency which was maintained at the levels observed in sham mice. Further investigation to elucidate specific T and B cell subset interactions is required to determine if this coordination is pro- or anti-inflammatory in our model.

We acknowledge that certain limitations exist in our study. First, sex differences were considered but not performed in these studies. We focused our studies here in male mice because most of our previous studies were conducted in male mice. We are aware from other studies using female mice that we must carefully assess the appropriate dosage of NMEG-CGRP to use in female mice prior to performing TAC. Furthermore, we need to establish the pathology of TAC surgery in a female mouse model, as it is known that heart failure with preserved ejection fraction is the predominant phenotype observed in women [59]. Additionally, several studies have shown that CGRP is modulated by estrogen and progesterone [60,61]. We plan to conduct future studies that investigate the use of these CGRP analogs in the treatment of cardiovascular diseases in our TAC model and others that reflect sex differences in clinical presentation and thoroughly investigate the interaction of sex hormones with CGRP. Additionally, we limited our assessment to a single strain of C57BL6/J mouse. We chose to use C57Bl6/J mice because it is the predominant strain used in TAC models of HF due to its robust hypertrophic response [62,63]. Lastly, although we measured the reduction in blood pressure and cardiac parameters, we did not measure the changes in aortic flow velocities induced by TAC. However, our results related to cardiac hypertrophy, fibrosis, apoptosis, and oxidative stress measurements align with other TAC studies where pressure gradients are recorded [64].

The significance of our findings lies in the possibility that NMEG-CGRP may have the ability to reverse cardiac remodeling in HF pathophysiology. Current HF therapeutics function as maintenance drugs that slow disease progression, and the few studies that report reverse remodeling are small in size and rely solely on ventricular function assessments [65]. The aim of our previous study was to develop a CGRP analog that could sustain its bioactivity long enough to prevent the development of cardiac dysfunction without requiring a continuous delivery system. After successfully demonstrating that NMEG-CGRP could prevent the onset of HF pathophysiology, we shifted our focus in this study to developing interventional models that determine therapeutic response after the onset of cardiac dysfunction. HF is often first diagnosed in an acute care setting when cardiac dysfunction has become significant, making the need for restorative therapeutics crucial [66]. Our findings support the necessity for additional investigation of α-CGRP and its analogs in the treatment of cardiovascular diseases. Here, we showed that NMEG-CGRP attenuates progressive cardiac remodeling through positive hemodynamic regulation and anti-hypertrophic, anti-fibrotic, and anti-inflammatory mechanisms. Additionally, we showed that NMEG-CGRP is cardioprotective after the establishment of LV dysfunction in pressure overload HF models. NMEG-CGRP has the potential to become a novel treatment that prevents progression in cardiovascular diseases driven by cardiac remodeling.

## Figures and Tables

**Table 1:** Antibodies and fluorophores used for flow cytometry.

Antibody	Fluorophore	Catalog Number
CD11c	Brilliant Violet 421™	117329 ^1^
CD4	Brilliant Violet 570™	100542 ^1^
I-A/I-E	Brilliant Violet 605™	107639 ^1^
CD192 (CCR2)	Brilliant Violet 650™	150613 ^1^
CD68	Brilliant Violet 711™	137029 ^1^
CD11b	Brilliant Violet 750™	101267 ^1^
CD117 (c-kit)	Brilliant Violet 785™	135138 ^1^
CD8a	PerCP/Cyanine5.5	155014 ^1^
Ly-6C	APC/Cyanine7	128026 ^1^
CD106	APC	105718 ^1^
CD31	Alexa Fluor 700	102444 ^1^
TER-119	FITC	116206 ^1^
NK1.1	Spark YG™ 581	156531 ^1^
CD34	PE/Dazzle™ 594	128615 ^1^
CD41	PE/Cyanine7	133916 ^1^
CD45	NovaFluor™ Yellow 730	M005T02Y07-A ^2^
CD3	BUV563	741235 ^3^
CD45R/B220	BUV661	612972 ^3^
CD19	BUV737	612781 ^3^
Ly-6G	BUV805	741994 ^3^

^1^ BioLegend, San Diego, CA, USA. ^2^ Invitrogen, Carlsbad, CA, USA. ^3^ BD Biosciences, San Jose, CA, USA.

## Data Availability

The original contributions presented in this study are included in the article/Supplementary Material. Further inquiries can be directed to the corresponding author.

## Supplementary Materials

The following supporting information can be downloaded at: https://www.mdpi.com/article/10.3390/cells14201580/s1, Table S1. Per-animal ejection fraction data values; Figure S1: Flow cytometry gating strategy; Figure S2: Analysis of elastin tortuosity; Figure S3: Changes to immune cell subsets over time.

## Abbreviations

The following abbreviations are used in this manuscript:

4-HNE	4-hydroxy-2-nonenal
8-OHdG	8-hydroxy-2′-deoxyguanosine
α-CGRP	alpha-calcitonin gene-related peptide
ANOVA	analysis of variance
BP	blood pressure
BSA	bovine serum albumin
cDCs	conventional dendritic cells
CGRP	calcitonin gene-related peptide
CMEG-CGRP	human C-terminal NMEG-α-CGRP
DABCO	1,4-diazabicyclo-2,2,2-octane
DAPI	4′,6-diamidino-2-phenylindole
DCs	dendritic cells
EDTA	ethylenediaminetetraacetic acid
FBS	fetal bovine serum
HBSS	Hank’s balanced salt solution
HF	heart failure
HW/TL	heart weight to tibia length ratio
IACUC	Institution Animal Care and Use Committee
IgG	immunoglobulin G
KO	knockout
LV	left ventricle
NIH	National Institute of Health
NMEG	N-methoxyethyl glycine
NMEG-CGRP	human N-terminal NMEG-α-CGRP
PBS	phosphate-buffered saline
PFA	paraformaldehyde
RBC	red blood cell(s)
RCF	relative centrifugal force
RT	room temperature
SEM	standard error of the mean
TAC	transverse aortic constriction
TUNEL	terminal deoxynucleotidyl transferase dUTP nick-end labeling
WGA	wheat germ agglutinin
