# Supplementary material for: A Novel Peptoid Hybrid of Alpha-Calcitonin Gene-Related Peptide (α-CGRP) Ameliorates Cardiac Remodeling in Pressure Overload-Induced Heart Failure
Source: Cells. 2025 Oct 11;14(20):1580. doi: 10.3390/cells14201580 (PMC12564457; doi:10.3390/cells14201580)
Supplement: Supplementary file 1 [file cells-14-01580-s001.zip › cells-3883506-supplementary.pdf]

Cells

Supporting Information for

A Novel Peptoid Hybrid of Alpha-Calcitonin Gene Related Peptide (α-CGRP) Ameliorates Cardiac Remodeling in Pressure Overload-Induced Heart Failure

Sarah Deloach, Ambrish Kumar, Emily Ruggiero, Ryan Ball, Kamryn Gleason, Jason Kubinak, Donald J. DiPette, and Jay D. Potts

Mouse ID	Treatment	D0	D9	D16	D23	D30	D37	D44
38.23	Sham	83.32	77.64	81.66	77.29	80.71	79.40	78.59
38.25	Sham	79.35	81.08	79.18	78.30	81.06	80.69	79.00
38.26	Sham	80.14	82.52	77.63	81.99	79.40	80.25	79.35
39.16	Sham	77.53	79.03	80.08	81.64	80.43	82.21	80.85
39.17	Sham	82.73	80.21	79.78	78.00	80.05	81.29	80.08
39.18	Sham	78.48	78.80	78.96	79.56	79.80	78.32	80.34
39.19	Sham	76.98	78.17	81.82	79.31	76.61	80.14	80.79
39.20	Sham	76.68	81.60	81.19	77.81	80.83	79.12	80.67
38.21	Sham+NMEG-CGRP	81.73	77.00	82.68	79.34	79.80	80.27	79.39
38.22	Sham+NMEG-CGRP	78.74	80.35	80.29	80.09	80.81	78.95	77.87
38.24	Sham+NMEG-CGRP	81.37	78.29	78.24	79.12	81.91	*	78.83
39.6	Sham+NMEG-CGRP	77.10	69.85	70.30	71.79	70.36	71.69	75.96
39.10	Sham+NMEG-CGRP	78.64	71.41	72.16	72.73	65.55	72.19	68.37
39.11	Sham+NMEG-CGRP	79.52	71.98	73.36	72.08	68.89	71.70	70.04
39.24	Sham+NMEG-CGRP	77.33	72.52	63.21	70.05	71.71	72.03	70.25
38.15	TAC	82.38	69.85	66.69	65.26	62.68	60.23	50.96
38.16	TAC	79.78	73.86	70.69	66.94	56.00	55.67	43.30
38.17	TAC	79.03	73.51	67.46	57.33	56.85	45.87	41.69
39.1	TAC	80.63	72.11	65.69	65.25	57.88	49.42	41.95
39.3	TAC	76.86	72.89	72.50	70.71	59.62	56.50	55.38
39.5	TAC	81.22	77.10	68.88	62.49	60.52	54.13	46.58
39.26	TAC	82.25	72.11	68.17	63.67	60.44	45.51	45.53
39.27	TAC	77.88	70.33	72.16	69.05	55.03	47.24	51.87
38.6	TAC+NMEG-CGRP	82.41	74.88	70.91	67.97	73.02	69.40	73.26
38.7	TAC+NMEG-CGRP	78.82	69.67	68.41	71.25	76.31	73.99	70.15
38.10	TAC+NMEG-CGRP	79.62	72.48	68.22	69.87	72.60	71.39	70.70
38.11	TAC+NMEG-CGRP	80.92	72.62	71.56	71.30	72.31	73.50	73.07
38.14	TAC+NMEG-CGRP	78.43	72.99	71.74	71.05	70.13	68.55	69.05
39.14	TAC+NMEG-CGRP	80.23	81.13	81.79	78.79	77.45	77.18	80.46
39.15	TAC+NMEG-CGRP	80.35	79.12	80.31	78.33	80.47	78.42	79.55

39.21	TAC+NMEG-CGRP	77.16	79.18	79.01	77.47	73.88	83.64	78.54
39.22	TAC+NMEG-CGRP	78.97	64.87	77.27	75.65	79.54	78.79	79.12

Table S1. Per-animal ejection fraction data values. Ejection fraction values measured per-animal at each timepoint across the experimental timeline. *Value could not be obtained.

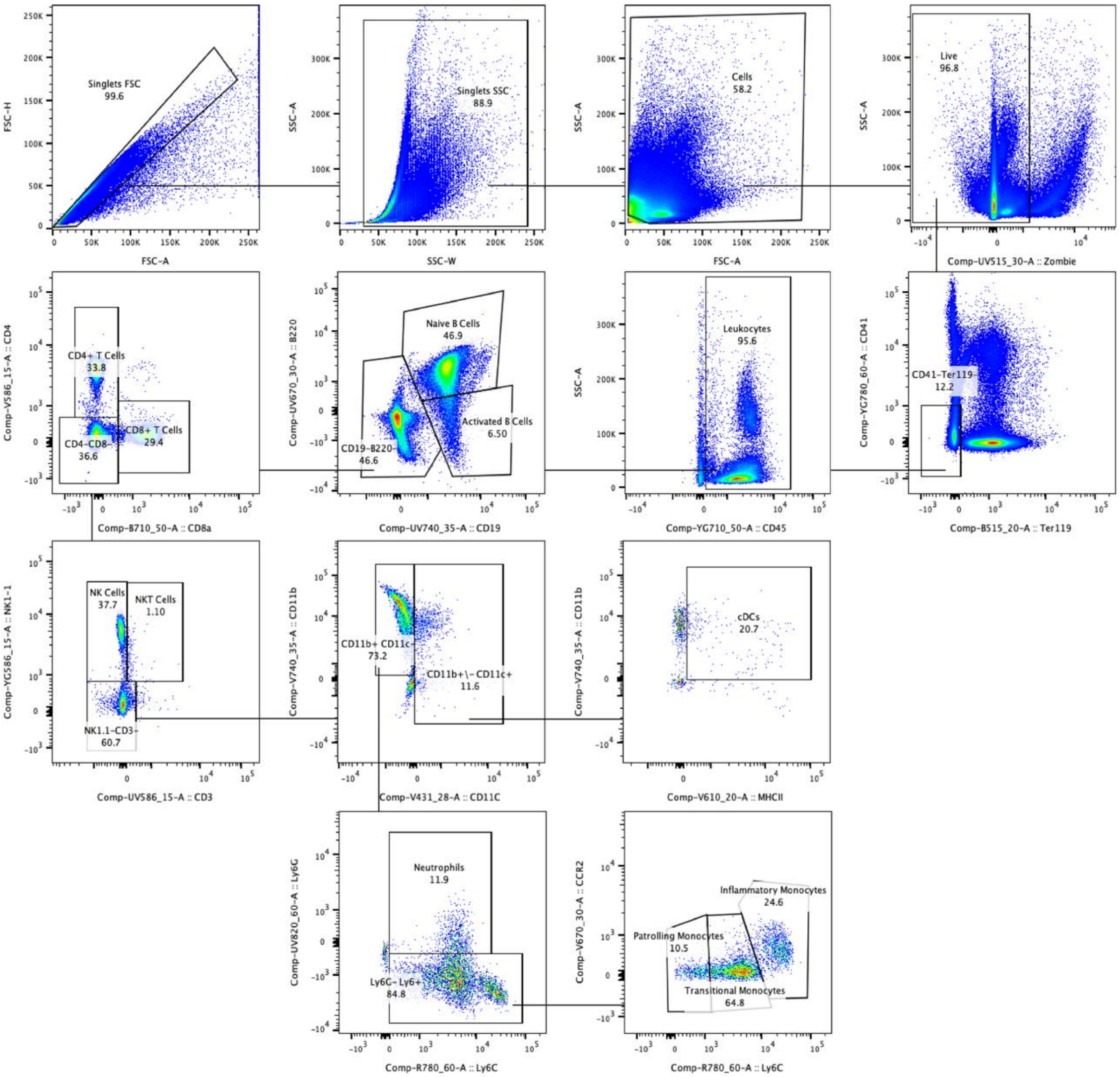

Figure S1. Flow cytometry gating strategy. Doublets were excluded with FSC-H vs. FSC-A and SSC-A vs. SSC-W gates. Debris was excluding with an SSC-A vs. FSC-A gate followed by dead cells with an SSC-A vs. ZombieAqua™ negative gate. Red blood cells and endothelial cells were excluded with a CD41 vs. Ter119 negative gate, and leukocytes were drilled down on with an SSC-A vs. CD45 positive gate. B220 vs. CD19 axes were used to isolate naïve and activated B cells, with naïve B cells identified as CD19+B220+/hi and activated B cells identified as CD19+B220-/lo. CD4 vs. CD8a axes were used to isolate CD4+ T cells and CD8+ T cells from CD19-B220- cells. NK1.1 vs. CD3 axes were used to isolate NK and NKT cells from CD4-CD8- cells. CD11b vs. CD11c axes were used to differentiate myeloid cell lineages with a CD11b+CD11c- gate and a CD11b+\-CD11c+ gate. Within the CD11b+\-CD11c+ gate, CD11b vs.

MHCII axes were used to identify conventional dendritic cells (cDCs) as CD11b+MHCII+. Within the CD11b+-CD11c- gate, Ly6G vs. Ly6C axes were used to isolate neutrophils and Ly6G-Ly6C+ populations, with neutrophils identified as Ly6G+Ly6C+. Within the Ly6G-Ly6C+ gate, monocyte subsets were identified using CCR2 vs. Ly6C axes. Patrolling monocytes were identified as CCR2-\lo Ly6C lo, transitional monocytes were identified as CCR2-\lo Ly6C+\int, and inflammatory monocytes were identified as Ly6C+/hi CCR2+.

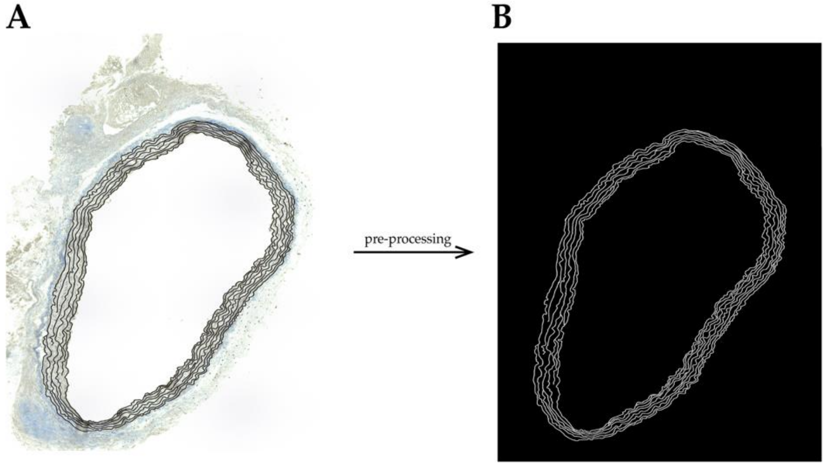

Figure S2. Analysis of elastin tortuosity. (A) Sections of the distal aortic arch were stained with modified Masson's Trichrome and imaged using a Revolve Microscope. Images were imported into Fiji for pre-processing. **(B)** Elastin fibers were manually traced using the paintbrush tool, followed by image thresholding and binarization.

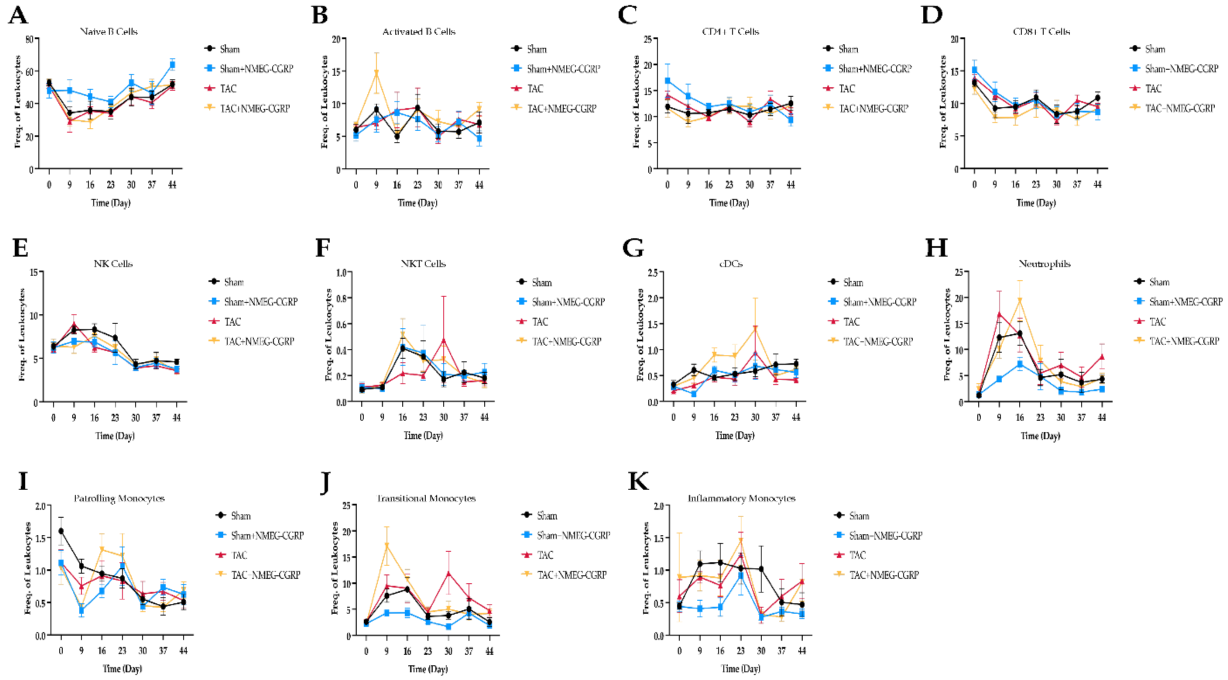

Figure S3. Changes to immune cell subsets over time. Mixed-effects analysis with main effects of time and treatment with time x treatment interactions across **(A)** naïve B cells, **(B)** activated B cells, **(C)** CD4+ T cells, **(D)** CD8+ T cells, **(E)** natural killer (NK) cells, **(F)** NKT cells, **(G)** conventional dendritic cells (cDCs), **(H)** neutrophils, **(I)**) patrolling monocytes, **(J)** transitional monocytes, and **(K)** inflammatory monocytes.
